# Chitin-Induced Airway Epithelial Cell Innate Immune Responses Are Inhibited by Carvacrol/Thymol

**DOI:** 10.1371/journal.pone.0159459

**Published:** 2016-07-27

**Authors:** Ali Reza Khosravi, David J. Erle

**Affiliations:** 1 Mycology Research Center, Faculty of Veterinary Medicine, University of Tehran, Tehran, Iran; 2 Lung Biology Center, Department of Medicine, University of California, San Francisco, California, United States of America; Universitatsklinikum Freiburg, GERMANY

## Abstract

Chitin is produced in large amounts by fungi, insects, and other organisms and has been implicated in the pathogenesis of asthma. Airway epithelial cells are in direct contact with environmental particles and serve as the first line of defense against inhaled allergens and pathogens. The potential contributions of airway epithelial cells to chitin-induced asthma remain poorly understood. We hypothesized that chitin directly stimulates airway epithelial cells to release cytokines that promote type 2 immune responses and to induce expression of molecules which are important in innate immune responses. We found that chitin exposure rapidly induced the expression of three key type 2-promoting cytokines, IL-25, IL-33 and TSLP, in BEAS-2B transformed human bronchial epithelial cells and in A549 and H292 lung carcinoma cells. Chitin also induced the expression of the key pattern recognition receptors TLR2 and TLR4. Chitin induced the expression of miR-155, miR-146a and miR-21, each of which is known to up-regulate the expression of pro-inflammatory cytokines. Also the expression of SOCS1 and SHIP1 which are known targets of miR-155 was repressed by chitin treatment. The monoterpene phenol carvacrol (Car) and its isomer thymol (Thy) are found in herbal essential oils and have been shown to inhibit allergic inflammation in asthma models. We found that Car/Thy inhibited the effects of chitin on type 2-promoting cytokine release and on the expression of TLRs, SOCS1, SHIP1, and miRNAs. Car/Thy could also efficiently reduce the protein levels of TLR4, inhibit the increase in TLR2 protein levels in chitin plus Car/Thy-treated cells and increase the protein levels of SHIP1 and SOCS1, which are negative regulators of TLR-mediated inflammatory responses. We conclude that direct effects of chitin on airway epithelial cells are likely to contribute to allergic airway diseases like asthma, and that Car/Thy directly inhibits epithelial cell pro-inflammatory responses to chitin.

## Introduction

Chitin is an essential component of the fungal cell wall and of the exoskeletons of crabs, shrimp, and insects and is a common constituent of house dust [1, 2]. In humans, elevated chitin exposure in the workplace and at home correlates with asthma and other allergic diseases [2–4]. In animal models of asthma, chitin administration induced type 2 immune responses, eosinophilic inflammation, and alternative macrophage activation [2, 5–8]. One lung cell type implicated in the response to chitin is the macrophage. Chitin stimulation can elicit production of IL-17A and TNF by macrophages via activation of the toll-like receptor (TLR) 2 [5, 6]. The airway epithelium is a first line of defense against inhaled particles and pathogens and is an important source of cytokines, including IL-25, IL-33, and TSLP, that promote type 2 immune responses [9, 10]. Van Dyken et al. reported that chitin particles induce inflammatory responses in lung and induce expression of IL-25, IL-33, and TSLP [11]. Roy *et al*. found that airway epithelial cells produce CCL2 (MCP-1) in response to chitin and this response is required for chitin-induced M2 polarization and allergic inflammation in vivo [12]. Other potential effects of chitin on airway epithelial cells remain unknown. However, the finding that chitin stimulation of keratinocytes resulted in increased secretion of CXCL8, IL-6, and TSLP and increased levels of TLR2 and TLR4 mRNA [7] suggests that direct effects of chitin on airway epithelial cells may modulate multiple pathways which are important in asthma.

Suppressor of Cytokine Signaling 1 (SOCS1) is a negative regulator of IL-4–dependent pathways in vitro. Therefore, it might control type-2 immunity such as IL-5 and IL-13 induction and eosinophilic mucosal inflammation, which are implicated in allergic asthma [13]. SH2 domain-containing inositol polyphosphate 5′ phosphatase 1 (SHIP1) is believed to be a negative regulator in a variety of cytokine, and growth factor signaling pathways in different cell types. SHIP1 deficient mice develop spontaneous Th2 inflammation in the lungs, demonstrating the importance of this molecule in controlling pulmonary immune responses [14].

miRNAs are a class of gene regulators which bind to the 3’ untranslated regions of target mRNAs and direct their post-transcriptional repression. miRNAs have emerged as important regulators of TLR signaling [15]. In addition, several miRNAs have been shown to be up-regulated in response to TLR ligands [16]. miR-155, miR-146a and miR-21 have important roles in the pathogenesis of Th2 responses in asthma and other inflammatory diseases [17]. SOCS1 and SHIP1 are two direct targets of miR-155 that are important in immune responses [18].

The plant-derived molecules carvacrol (Car) and thymol (Thy) have been shown to have anti-inflammatory activity in asthma and other disease models but the basis of these effects is incompletely understood. Car and its isomer Thy are major monoterpenic phenols found in essential oils from aromatic plants. These bioactive compounds are known to possess an extensive variety of pharmacological properties such as antioxidant, antimicrobial, anti-cancer and anti-inflammatory activities [19–21]. In guinea pig models of allergic asthma, administration of Car inhibited inflammation, tracheal hyper-reactivity and production of the type 2 cytokine IL-4 [22]. Car/Thy has been reported to have effects on a variety of cell types, including T cells, myeloid cells, and endothelial cells [19], but the possibility that Car/Thy may have direct effects on airway epithelial cells has not been addressed previously.

In the present study, the first goal was to investigate whether chitin induces release of type 2 promoting cytokines and affects other epithelial cell pathways which are important in innate immune responses. The second goal was to investigate whether Car/Thy affects epithelial cell responses to chitin.

## Materials and Methods

### Preparation of Chitin and Car/Thy

Chitin was derived from *Mucor rouxii* (ATCC 24905). Chitin particles were purified as previously described [23, 24]. Mean chitin particle size was ~40 μm. Chitin pellets were separated, lyophilized and resuspended at a final concentration of 80 μg/ml based on a previous report [12]. A mixture of Car and Thy (74% and 26%, respectively) was isolated from *Origanum vulgare*, as previously described [21]. We used Car/Thy at dose of 200 μM (equal to 30 μg/mL) which had no effects on cell viability (S1 Fig) [25, 26]. To test for microbial contamination aliquots of the samples were incubated in LB broth. Chitin, Car/Thy and media each had endotoxin levels ≤ 0.1 EU/ml as determined using the Limulus Amebocyte Lysate (LAL) kit (QCL-1000, Lonza Group Ltd., Basel, Switzerland).

### MTT assay

Cells were cultured in RPMI 1640 (Gibco-BRL, Grand Island, NY, USA) without phenol red and treated for 24 h with chitin or Car/Thy at concentrations ranging from 50 to 1000 μg/mL. MTT (3-[4,5-dimethylthiazol-2-yl]-2,5-diphenyltetrazolium bromide; thiazolyl blue, Sigma-Aldrich, Munich, Germany, Cat. No. M5655) was added to wells containing cells at a final concentration of 0.5 mg/mL. Enzymatic conversion of MTT to formazan was detected by for colorimetric analysis at a wavelength of 570 nm (BioTek ELx808, BioTek Instruments, Inc. Winooski, VT, USA). All tests were done in triplicate. Cells treated with Triton-X100 (1%, Sigma-Aldrich, Munich, Germany, Cat. No. T8787) were used as positive controls.

### Cell growth conditions and treatment

Beas-2B epithelial cells from normal human bronchial epithelium, A549 human alveolar adenocarcinoma cells, and H292 human muco-epidermoid lung carcinoma cells were cultured in DMEM (Gibco-BRL, Grand Island, NY, USA) supplemented with 10% fetal bovine serum (FBS, Gibco-BRL, Grand Island, NY, USA), penicillin (50 IU/ml), and streptomycin (50 μg/ml) (Gibco-BRL, Grand Island, NY, USA) and incubated at 37°C in 5% CO2. The number of viable cells was determined by trypan blue exclusion. 1 x 10^6^ cells were plated and grown overnight in 3 ml of medium in 6-well plates prior to cytokine and mRNA assay. The DMEM was aspirated out from the cultures and fresh DMEM without serum containing the indicated concentrations of Car/Thy or chitin was added into the plates. Also, Car/Thy was added together with chitin as indicated. Poly (I:C) and LPS were added to the cultured cells in separate wells as positive controls. Triplicate cultures were used for each condition. The number of viable cells after 24 h exposure to each of the treatments was determined by trypan blue exclusion.

### Real-Time Quantitative PCR

Total RNA was extracted from cell cultures using the RNeasy mini-kit (Qiagen GmbH, Hilden, Germany), according to the manufacturer’s instructions. RNA was eluted from column to into 50 μL nuclease-free water and was stored at -80°C until use. RNA concentration and quality were measured using a Nanodrop system (NanoDrop Technology, San Diego, CA, USA). Reverse transcription of total RNA was done using iScript cDNA synthesis kit (Bio-Rad, Hercules, CA, USA) according to the manufacturer’s protocol. Real time PCR was carried out using the SYBR Green method using following primers: TLR2: Forward: TGCTGCCATTCTCATTCTTCTG, Reverse: AGGTCTTGGTGTTCATTATCTTCC, TLR4: Forward: CAACCAAGAACCTGGACCTG, Reverse: GAGAGGTGGCTTAGGC, SOCS1: Forward: CACCTTCTTGGTGCGCG, Reverse: AAGCCATCTTCACGCTGAGCTCTG, SHIP1: Forward: GCGTACACCAAGCAGAAAGC, Reverse: GGACCGTTCTTGGAGACAAA, GAPDH: Forward: CCACTCCTCCACCTTTGACG, Reverse: CCACCACCCTGTTGCTGTAG.

qPCR data were analyzed by the delta delta CT (ddCT) method [27] and normalized to GAPDH. For miRNAs, reverse transcription of total RNA was done using TaqMan specific RT primers and reverse transcription kits (Applied Biosystems, Foster City, CA, USA). Quantitative real time PCR was performed according to the manufacturer’s protocol using an ABI 7900HT system (Applied Biosystems, Foster City, CA, USA). Raw threshold cycle values were normalized using RNU-6B (Cat#4427975, Thermo Fisher Scientific, San Jose, CA, USA) as the internal control.

### Measurement of cytokines

Levels of IL-25, IL-33 and TSLP in supernatants were assessed using ELISA kits (R&D systems, Minneapolis, MN., USA). The data are presented as mean ± SD of triplicate assays.

### Western Blot

Cells were washed twice with PBS. Membrane and cytosol proteins were extracted according to the manufacturer’s instructions using the Qproteome Cell Compartment Kit (Qiagen, Hilden, Germany, Cat. No. 37502). Protein concentration was measured using the Bradford method. Extracted proteins were resolved in sodium dodecyl sulphate–polyacrylamide (SDS) gel electrophoresis under reducing conditions and then transferred to nitrocellulose. The blocking was done using TBST buffer (25 mm Tris-HCl, pH8·0, 125 mm NaCl, 0·1% Tween 20) containing 5% fat-free milk. Blots were incubated with anti-Actin, anti-TLR2, anti-TLR4, anti-SOCS1, anti-SHIP1 antibodies (Santa Cruz Biotechnology, Santa Cruz, CA, USA) and horseradish peroxidase (HRP)-conjugated IgG. The membranes were visualized using SuperSignal West Pico Chemiluminescent Substrate (Thermo Fisher Scientific, San Jose, CA, USA, Cat. No. 34080).

### Statistical analysis

MedCalc version 12 and GraphPad Prism version 6 were used for statistical analysis. All experiments used triplicate cultures and material from each culture was analyzed in triplicate (e.g., three ELISAs or PCRs from each of three cell cultures). The normality of data was checked by Kolmogorov-Smirnov test. Non-linear three parametric regression analysis was done to calculate IC50 for chitin and Car/Thy. One-way ANOVA was done to test the significance across all time-points and Dunnett’s post-hoc test was used to test observed differences between each time point and the control group. Tukey’s *HSD* post-hoc test was done to test differences between chitin- and chitin plus Car/Thy-treated cells at each time-point. Bands densities from western blots were measured using ImageJ software version 1.49 [28]. *P* values *<* 0.05 were considered as significant. The data were presented as means ± SD.

## Results

### Chitin stimulated release of type-2 promoting cytokines from airway epithelial cells is suppressed by Car/Thy

**Fig 1:**
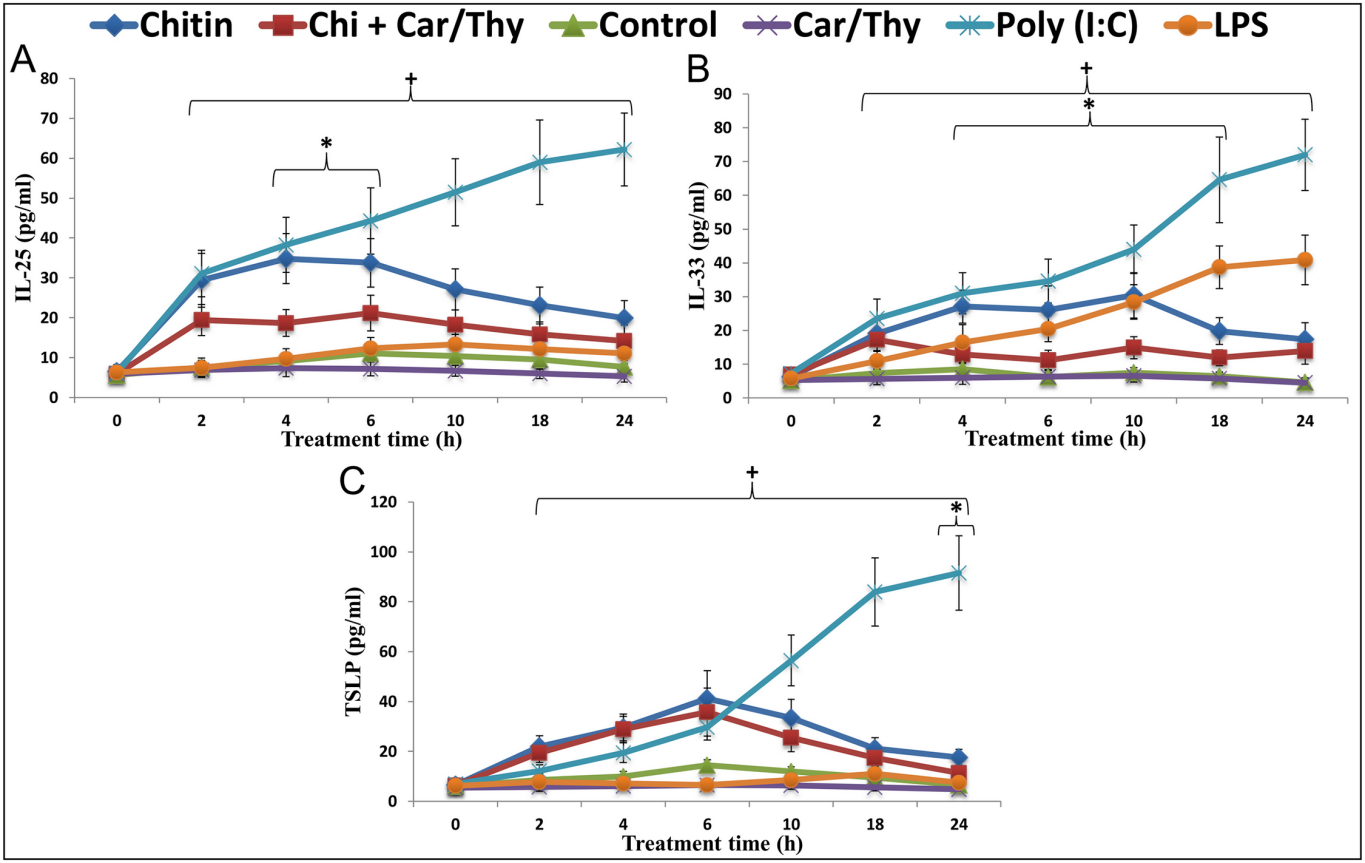
IL25, IL33 and TSLP ELISA levels in Chitin- and Chitin plus Car/Thy –treated Beas-2B cells. Levels of IL-25 (A), IL-33 (B), and TSLP (C) in supernatant from control, LPS, poly (I:C), Car/Thy, chitin, and chitin plus Car/Thy-treated cells were determined by ELISA. *, *p* < 0.05 for the comparison between Chitin and Chitin plus Car/Thy at the same time point by Tukey’s HSD test. +, *p* < 0.05 for the comparison between Chitin-treated and control cells by Dunnett’s test.

We stimulated BEAS-2B human bronchial epithelial cells with chitin and measured the release of IL-25, IL-33, and TSLP. We used a concentration of chitin which was similar to concentrations used in previous studies of other cell types [29, 30] and did not impair cell viability (S1 Fig). We used Poly (I:C) and LPS as positive controls. Poly (I:C) could strongly stimulate the release of IL-25, IL-33 and TSLP in BEAS-2B cells (*p* < 0.05, Dunnett’s test). LPS could only induce the release of IL-33 (*p* < 0.05, Dunnett’s test). Chitin treatment led to increases in levels of all 3 cytokines within 2 h, and levels remained elevated for at least 24 h. In BEAS-2B cells co-treatment with Car/Thy reduced chitin-stimulated IL-25 and IL-33 release but had very modest effects on TSLP (Fig 1). Similar effects of chitin and Car/Thy on IL-25, IL-33, and TSLP were seen with H292 human lung mucoepidermoid carcinoma cells and A549 human lung carcinoma cells (S2 Fig).

### Chitin-stimulated induction of TLR2 and TLR4 is suppressed by Car/Thy

**Fig 2:**
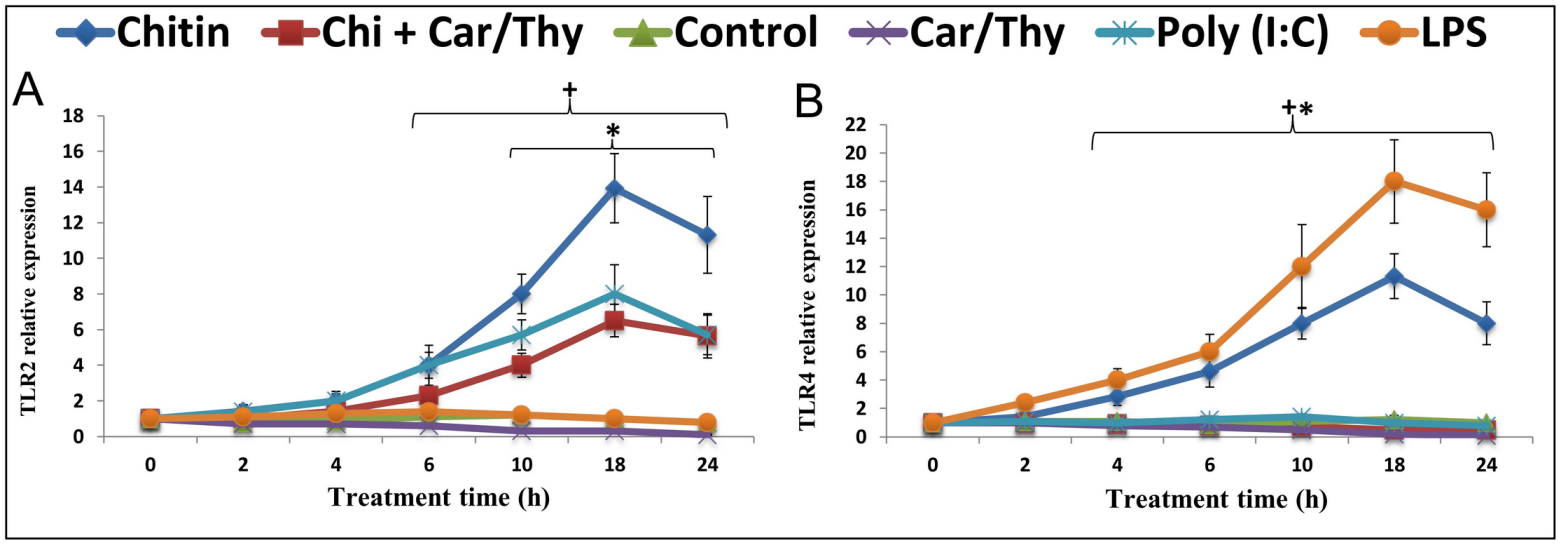
TLR2 and TLR4 expression in Chitin- and Chitin plus Car/Thy –treated Beas-2B cells. Levels of TLR2 (A) and TLR4 (B) mRNAs in control cells and LPS-treated, poly (I:C)-treated, Car/Thy-treated, chitin-treated or chitin plus Car/Thy-treated cells were determined by qRT-PCR. *, *p* < 0.05 for the comparison between Chitin and Chitin plus Car/Thy at the same time-point by Tukey’s HSD test. +, *p* < 0.05 for the comparison between Chitin-treated and control cells by Dunnett’s test.

TLR2 and TLR4 mRNAs were significantly up-regulated after chitin stimulation of BEAS-2B cells. Peak TLR2 and TLR4 expression were 13.9- and 9.4-fold higher than control cells, respectively at 18 h after the chitin treatment (Fig 2). Car/Thy treatment partially prevented the induction of TLR2 and completely prevented the induction of TLR4. Very similar results were obtained with H292 cells and A549 cells (S3 Fig). LPS as positive control could strongly induce the TLR4 expression but had minimal effect on TLR2 mRNA level. Inversely, Poly (I:C) could increase the TLR2 mRNA level but had no significant effect on TLR4 expression.

**Fig 3:**
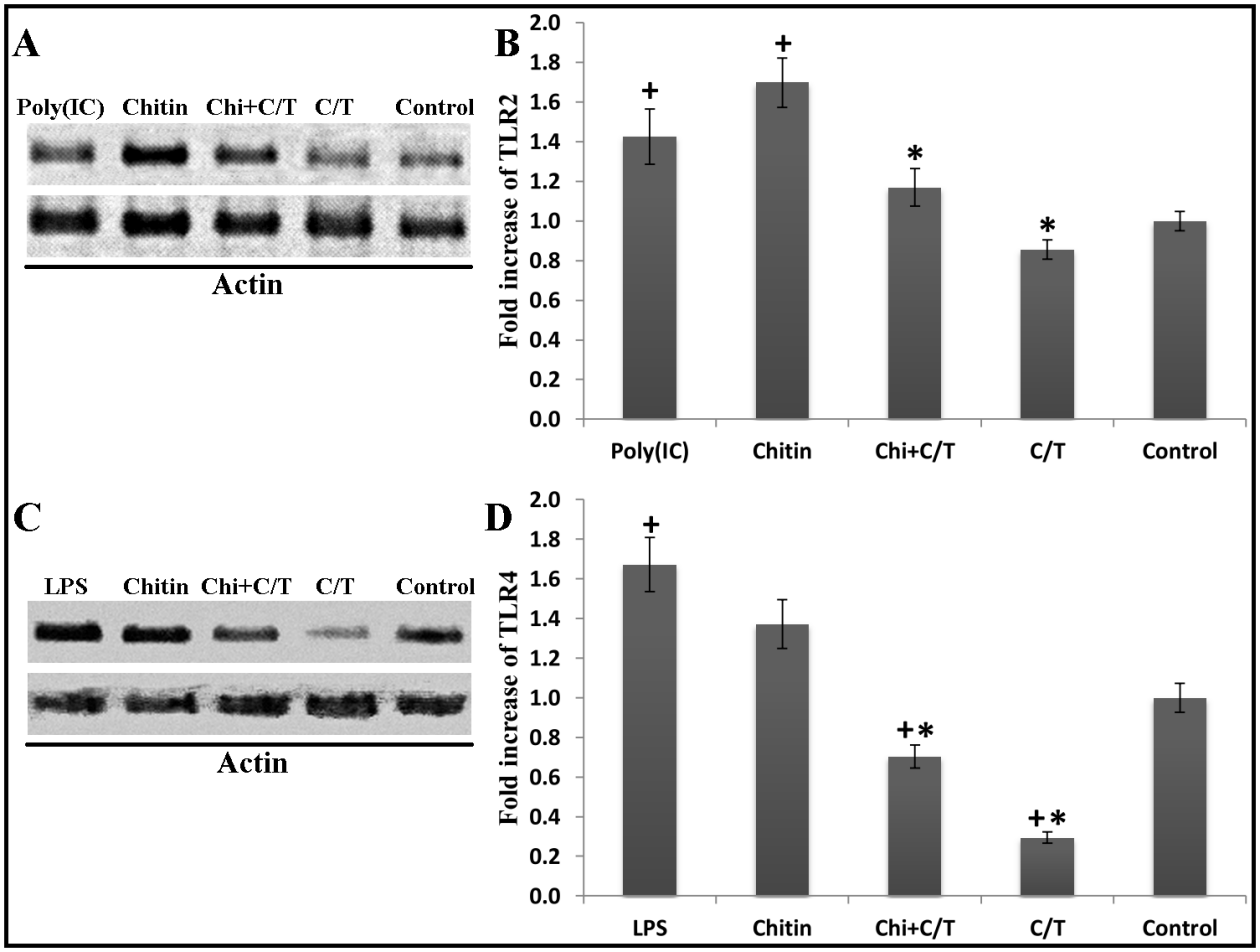
Protein levels of TLR2 and TLR4 in Chitin- and Chitin plus Car/Thy –treated Beas-2B cells after 24 h exposure. Western blot of TLR2 protein (A) and TLR2 protein fold increase (B) in untreated control cells, poly (I:C)-treated, chitin-treated and chitin plus Car/Thy-treated cells. Western blot of TLR4 protein (C) and TLR4 protein fold increase (D) in untreated control cells, LPS-treated, chitin-treated and chitin plus Car/Thy-treated cells. *, *p* < 0.05 for the comparison between Chitin and Chitin plus Car/Thy by Tukey’s HSD test. +, *p* < 0.05 for the comparison between Chitin-treated and control cells by Dunnett’s test. Bars represent mean ± standard deviation (SD).

TLR2 protein in BEAS-2B cells increased 1.9-fold after 24 h exposure to chitin (*p* < 0.05, Dunnett’s test) (Fig 3). TLR2 protein levels in chitin plus Car/Thy-treated cells were also significantly lower in than chitin-treated cells (*p* < 0.05, Tukey’s HSD test) (Fig 3). TLR4 protein increased 1.9-fold after 24 h exposure to chitin (*p* < 0.05, Dunnett’s test) (Fig 3). The TLR4 protein level in chitin plus Car/Thy-treated cells was also significantly lower than in chitin-treated cells (*p* < 0.05, Tukey’s HSD test) (Fig 3). Interestingly, levels of both TLR2 and TLR4 protein in chitin plus Car/Thy-treated cells were nearly similar to the levels in control cells. The protein levels of LPS- and Poly (I:C)-treated cells were consistent with mRNA results. Poly (I:C) and LPS increased the protein levels of TLR2 and TLR4 (43% and 67%, respectively).

### Chitin-induced increases in miR-155, miR-146a and miR-21 are suppressed by Car/Thy

**Fig 4:**
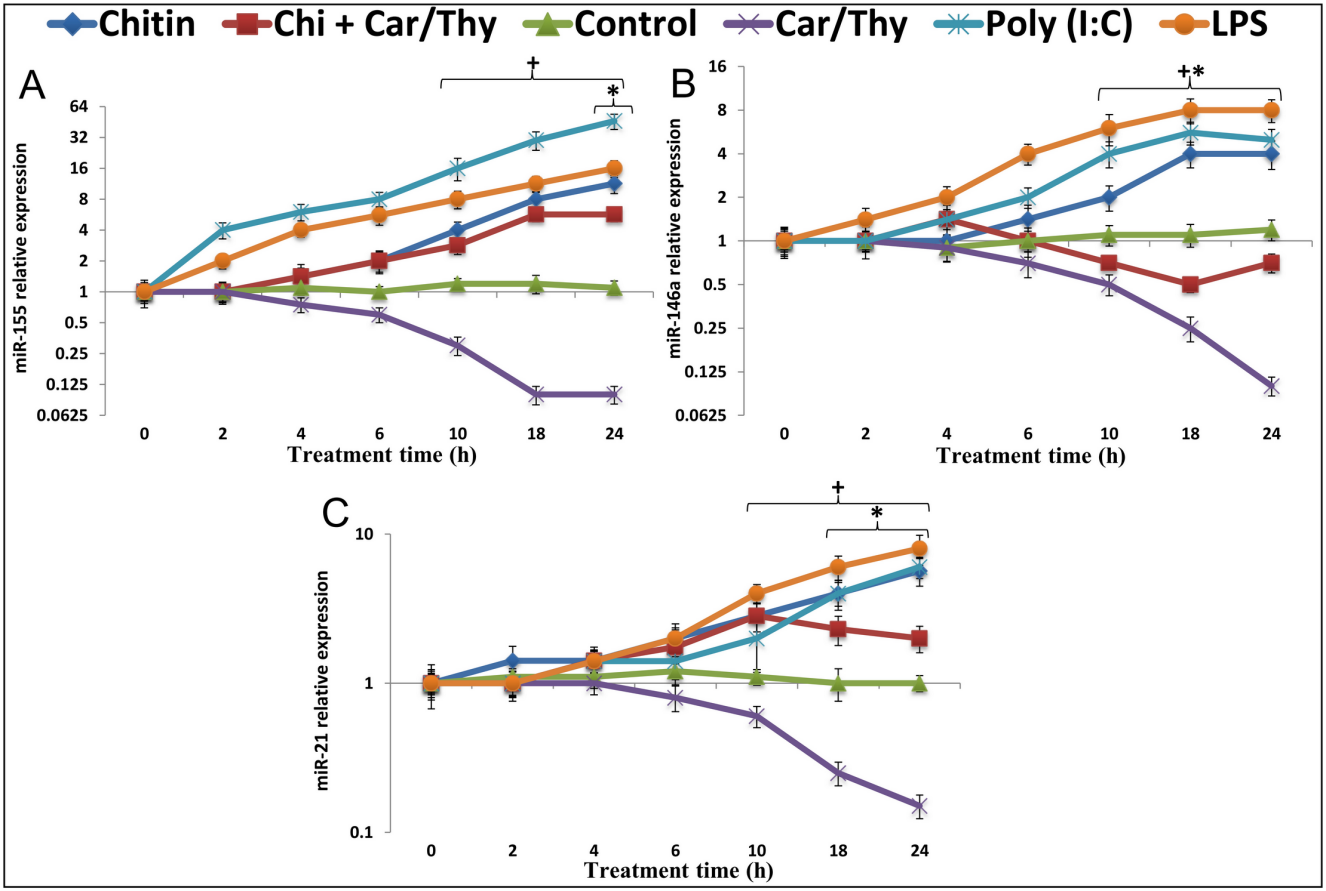
Expression of miR-155, miR-146a and miR-21 in Chitin- and Chitin+Car/Thy–treated Beas-2B cells. The expression levels of miR-155 (A), miR-146a (B) and miR-21 (C) in control cells and LPS-treated, poly (I:C)-treated, Car/Thy-treated and chitin-treated or chitin plus Car/Thy-treated cells were determined by qRT-PCR. *, *p* < 0.05 for the comparison between Chitin and Chitin plus Car/Thy at the same time-point by Tukey’s HSD test. +, *p* < 0.05 for the comparison between Chitin-treated and control cells by Dunnett’s test. Data were presented as means ± standard deviation (SD) in logarithmic scale.

miR-155, miR-146a and miR-21 were significantly up-regulated after chitin stimulation of BEAS-2B cells (peak increases ranging from 4- to 11.3-fold at 18–24 h, Fig 4). Car/Thy treatment partially prevented induction of miR-155 and miR-21 and completely prevented the induction of miR-146a. Both LPS and Poly (I:C) could induce the up-regulation of miR-155, miR-146a and miR-21. Poly (I:C) induced miR-155 64-fold after 24 h. LPS up-regulated levels of both miR-146a and miR-21 up to 8-fold after 24 h. Very similar results were obtained with H292 cells and A549 cells (S4 Fig).

### The miR-155 targets SHIP1 and SOCS1 are decreased following chitin stimulation

**Fig 5:**
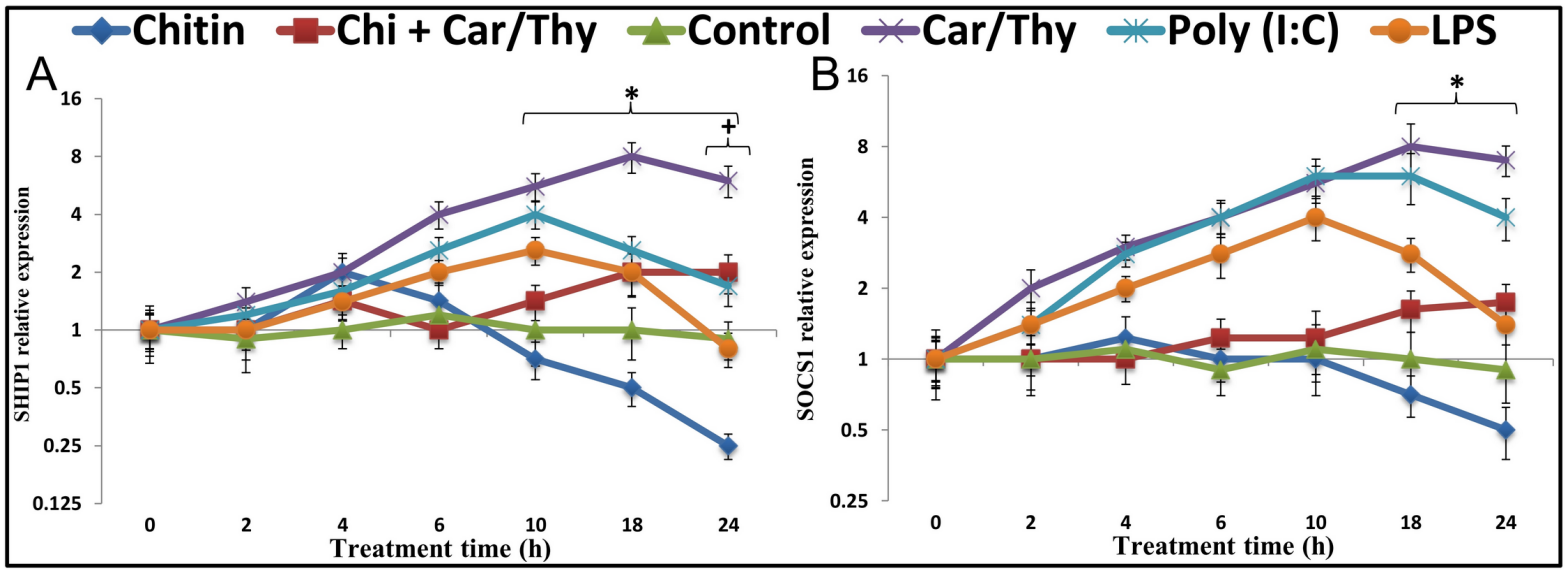
SHIP1 and SOCS1 relative expression in Chitin- and Chitin plus Car/Thy- treated Beas-2B cells. Expression levels of miR-155 (A), miR-146a (B) and miR-21 (C) in control cells and LPS-treated, poly (I:C)-treated, Car/Thy-treated and chitin-treated or chitin plus Car/Thy-treated cells were determined by qRT-PCR. *, *p* < 0.05 for the comparison between Chitin and Chitin plus Car/Thy at the same time point by Tukey’s HSD test. +, *p* < 0.05 for the comparison between Chitin-treated and control cells by Dunnett’s test. Dots on the graph represent means ± standard deviation (SD) in logarithmic scale.

SHIP1 decreased significantly after chitin stimulation of BEAS-2B cells (3.6-fold decrease at 24 h, Fig 5). SOCS1 reached its lowest expression level (1.8-fold decrease, not significant) at 24 h after chitin stimulation (Fig 5). Car/Thy treatment increased SHIP1 and SOCS1 mRNA to levels above those seen in untreated cells. Both LPS and Poly (I:C) induced up-regulation of SHIP1 and SOCS1 at the early time-points of stimulation but the levels were still lower than in Car/Thy-treated cells. The up-regulation was continued up to 10 h but then the mRNA levels reduced. At the last time-point (24 h) of exposure to LPS, the SHIP1 expression reached to a level lower than normal level but SHIP1 mRNA levels in Poly (I:C)-treated cell were remained up-regulated relative to normal levels. The same pattern of early up-regulation and late down-regulation was observed for SOCS1 after stimulation by LPS and Poly (I:C). The stimulation by Poly (I:C) at three time-points including 4, 6 and 10 h was as strong as the stimulation by Car/Thy (Fig 5). Very similar results were obtained with H292 cells and A549 cells (S5 Fig).

**Fig 6:**
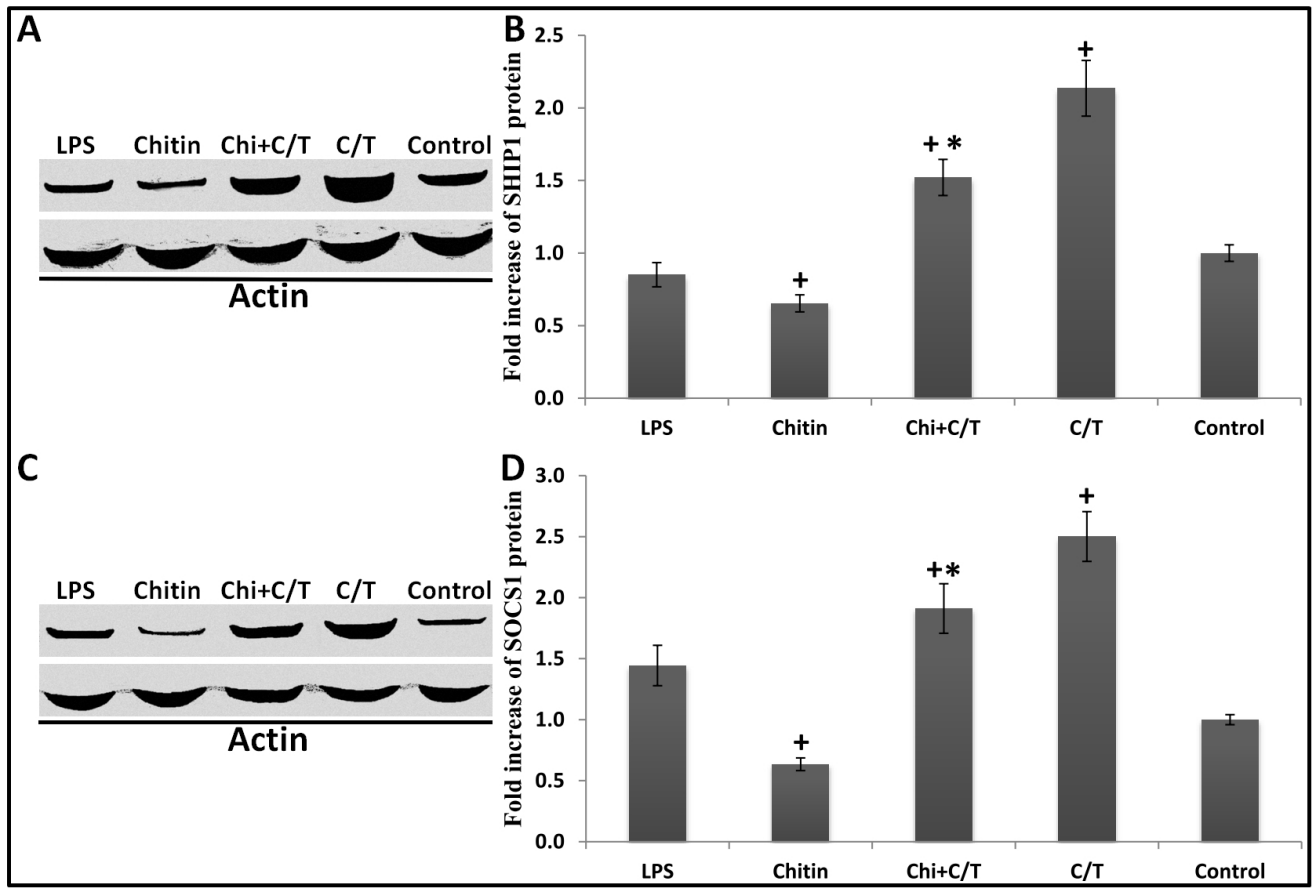
SHIP1 and SOCS1 protein levels in Chitin- and Chitin plus Car/Thy- treated Beas-2B cells. Western blot of SHIP1 protein (A) and SHIP1 protein fold increase (B) in untreated control cells and in cells treated with poly (I:C), chitin or chitin plus Car/Thy for 24 h. Western blot of SOCS1 protein (C) and SOCS1 protein fold increase (D) in untreated control cells, LPS-treated, chitin-treated and chitin plus Car/Thy-treated cells. In panel B and D, *, *p* < 0.05 for the comparison between Chitin and Chitin plus Car/Thy by Tukey’s HSD test. +, *p* < 0.05 for the comparison between treated cells (with LPS, chitin, chitin plus Car/Thy or Car/Thy) and control cells by Dunnett’s test. Experiments to detect SHIP1 and SOCS1 proteins were done in triplicate. Bars represent mean ± standard deviation (SD).

SHIP1 protein levels in chitin-treated and LPS-treated cells were lower than that in control cells. Both LPS and Chitin reduced SHIP1 in treated cells after 24 h of exposure, although the effect of LPS was smaller (15% reduction) and did not reach the statistical significance (*p* > 0.05, Dunnett’s test). SHIP1 protein level in chitin-treated cells reduced 35% in comparison to control cells (*p* < 0.05, Dunnett’s test). Car/Thy treatment increased SHIP1 protein levels when given alone and when given in combination with chitin. The increase in SHIP1 protein level was only 52% when cells were treated with a combination of chitin with Car/Thy whereas the increase was 214% in cells treated with only Car/Thy (Fig 6A and 6B). SOCS1 protein level increased 250% after 24 h exposure to Car/Thy in comparison to untreated (control) cells (*p* < 0.05, Dunnett’s test). Car/Thy treatment increased the SOCS1 protein levels when given alone and when given in combination with chitin (Fig 6C and 6D). Also, 24 h LPS-treatment increased the SOCS1 protein 44% compared with in untreated cells. Chitin-treatment strongly inhibited the SOCS1 protein in comparison to normal cells (37% reduction, *p* < 0.05, Dunnett’s test).

## Discussion

Airway epithelial cells are in direct contact with the environment. They not only act as a physical barrier and in mucocilliary transport but also have immunological functions. Activation of airway epithelial cells results in production of substances such as cytokines, chemokines and damage-associated molecular pattern molecules (DAMPs) to initiate innate and adaptive immune responses [10]. We studied the effects of chitin particles on A549, H292 and Beas-2B cells. Chitin, a long chain polymer of N-acetylglucosamine, is the second most abundant polysaccharide in nature after cellulose [1]. Chitin is distributed in the cell walls of organisms that are implicated in asthma [1, 31–34]. We found that chitin induced the type 2-promoting epithelial cytokines, IL-25, IL-33 and TSLP. Some previous reports [11, 35, 36] found increases in these type 2-promoting cytokines or their receptors in mouse lung after chitin administration and our results indicate that these findings can be explained by direct effects of chitin on epithelial cells and are relevant for humans. We also found that chitin affected the expression of other genes and miRNAs which are important in inflammatory responses. Our work complements a very recent study on mice which revealed that alveolar type II cells are a major source of IL-33 and TSLP which are produced in response to intranasal instillation of chitin, and that IL-33 and TSLP induce an interferon regulatory factor 4 (IRF4)-IL-9 pathway in ILC2s and enhance the production of IL-5 and IL-13 [37].

TLRs recognize pathogen-associated molecular patterns (PAMPs) to activate host defense against pathogens [38]. Our finding that chitin induces TLR2 and TLR4 suggests that a chitin-bearing organism might modulate immune responses by up-regulating TLR2 and TLR4 pathways. Several studies demonstrated that miRNAs can modulate allergic inflammation in asthma models [39, 40]. We focused on miR-21, miR-146a and miR-155, which have known roles in both innate and adaptive immune responses [39, 41]. We discovered that the expression of miR-155, miR-146a and miR-21 miRNAs were increased significantly in cells challenged with chitin. The expression of miR-21 increased in several models of asthma, although the expression was localized primarily to myeloid cells [42]. The roles of epithelial miR-21 in asthma are uncertain, although a previous report indicates that miR-21 targets mRNA for MARCKS [43], a known regulator of airway mucin secretion [44]. miR-146a directly targets several important immune signaling pathway molecules including IL-1 receptor-associated kinase 1 (IRAK1), TNF receptor-associated factor 6 (TRAF6), RelB and CARD10 [39, 45]. miR-155 regulates immune responses through effects on myeloid and lymphoid cells [46] but is also expressed in epithelial cells. We showed that chitin–induced increases in miR-155 were accompanied by decreases in two known targets: SOCS1 and SHIP1. We suggest that this miRNA may contribute to chitin-induced gene expression changes. Suppression of SHIP1 by miR-155 causes the activation of Akt kinase and up-regulation of IFN genes during the cellular response to LPS [47–49]. Some essential components of medicinal plants possess antimicrobial, anti-inflammatory and anti-cancer activity [21]. Car and Thy are major phenolic agents in some medicinal plants. Our results showed Car/Thy has suppressive effects on cytokine protein induction in epithelial cells challenged with chitin. The effect of chitin on the expression of TLR4 and TLR2, miRNA-21, miR-146a, miR-155, SHIP1 and SOCS1 were also inhibited by Car/Thy. Furthermore, our results revealed that in cells treated only by Car/Thy and not stimulated by chitin, the mRNA level of TLR2 and TLR4 and the levels of the miRNAs miR-155, miR-146a, miR-21 were decreased while mRNA levels of SHIP1 and SOCS1 were increased. The protein results were consistent with the mRNA expression data. These results indicate that Car/Thy could strongly reduce the activation of genes involved in initiating the inflammatory responses to levels even lower than in non-treated conditions. It has been previously shown in other cells that both Car and Thy can affect pathways that are associated with inflammatory responses and possess anti-inflammatory effects [50, 51]. Our results revealed novel pathways such as TLRs, miRNAs, SHIP1 and SOCS1 affected by Car/Thy. However, more studies are required to determine the precise mechanism of action of Car and Thy. Further studies would also be required to test whether miR-155 contributes to chitin- and Car/Thy-induced SHIP and SOCS1 gene expression changes and identify other pathways that may be important.

Based on our findings, we speculate that direct effect of Car/Thy on epithelial cells might inhibit the development of pathologic type 2 immune responses in human airways. In support of the general idea that compounds derived from essential oils of plants may have beneficial effects in asthma, previous work shows benefits of oral administration of 1.8-cineol [52], the major monoterpene of eucalyptus oil, and of inhalation of L-menthol [53, 54], a monocyclic compound from peppermint oil, in subjects with this disease. Therapeutic use of Car/Thy would require further in vivo studies to address efficacy, dose and route of administration, potential toxicity, pharmacokinetics and pharmacodynamics. The major monoterpenoid hydrocarbon found in native Iranian *Origanum vulgare* is carvacrol and thymol is the second most abundant monoterpene [55]. In this study we used a 3:1 mixture of carvacrol with thymol since some plants such as *Origanum vulgare* are rich in both carvacrol and thymol and since it was shown that a mixture of these two agents is less toxic to mammalian cells [56]. Rat oral LD50 for Thy and Car are 980 mg/kg and 810 mg/kg, respectively while the LD50 for combination of Car with Thy is 4,700 mg/kg [57]. As an alternative to oral delivery, the airway epithelium is well suited for targeting by inhalational delivery. A possible issue here is that both carvacrol and thymol are phenolic compounds and may have sensitization and irritant effects on skin or mucosal surfaces when applied by direct contact [57].

The concentrations of Car/Thy and chitin that we used had biological activity but do not appear to be cytotoxic. Previous studies showed that the significant reduction in viability of a human cell line (Caco-2) after 48 h treatment with Car was observed only at a concentration of 500 μM and that Thy had no effect on the viability of cells in concentrations up to 250 μM [26]. In addition, in Hep2 cells exposed to chitin for 24 h the IC50 of chitin determined by trypan blue exclusion assay was 400 μg/mL. The trypan blue exclusion assay showed that 50% of Hep2 cells were not viable/dead at a concentration of 400 μg/mL of chitin [29]. In the present study the IC50 of chitin determined by MTT assay for Beas-2B, H292 and A549 were 480.5, 305 and 176.7 μg/mL, respectively. Moreover, the IC50 of Car/Thy determined by MTT assay for Beas-2B, H292 and A549 were 576, 407.6 and 434 μg/mL, respectively. We used a mixture of Car/Thy at a concentration of 200 μM (equal to 30 μg/mL) and chitin at a concentration of 80 μg/mL for no more than 24 h. These concentrations were much lower than IC50s for chitin and Car/Thy. The number of viable cells determined by trypan blue exclusion assay was not significantly affected in cells treated with a combination of Car/Thy and chitin after 24 h exposure. The absence of detectable reduction in viability of treated cells and the normal morphology of treated epithelial cells also indicated that these treatments did not cause cell death (S1 Fig).

## Supporting Information

S1 Fig Chitin and Car/Thy cytotoxicity in Beas-2B, H292 and A549 cells. Viability of cells treated with Car/Thy (A) and chitin (B) at 50–1000 μM and 50–1000 μg/mL, respectively were determined by MTT assay after 24 h incubation. +, X and *, *p* < 0.05 are for the comparison between control cells and BEAS-2B, H292 and A549 cells, respectively by Dunnett’s test. Nearly 100% of cells treated with Triton-X100 (as positive control) were dead (not viable).
(TIF)

S2 Fig IL25, IL33 and TSLP ELISA levels in Chitin- and Chitin plus Car/Thy –treated H292 and A549 cells. Levels of IL-25 (A), IL-33 (B), and TSLP (C) in H292 cells and levels of IL-25 (D), IL-33 (E), and TSLP (F) in A549 cells in supernatant from control, LPS-treated, poly (I:C)-treated, Car/Thy-treated, chitin-treated and chitin plus Car/Thy-treated cells were determined by ELISA. *, *p* < 0.05 for the comparison between Chitin and Chitin plus Car/Thy at the same time point by Tukey’s HSD test. **+**, *p* < 0.05 for the comparison between Chitin-treated and control cells by Dunnett’s test.
(TIF)

S3 Fig TLR2 and TLR4 expression in Chitin- and Chitin plus Car/Thy –treated H292 and A549 cells. mRNA Levels of TLR2 (A) and TLR4 (B) in H292 cells and TLR2 (C) and TLR4 (D) in A549 cells in control cells and LPS-treated, poly (I:C)-treated, Car/Thy-treated, chitin-treated and chitin plus Car/Thy-treated cells were determined by qRT-PCR. *, *p* < 0.05 for the comparison between Chitin and Chitin plus Car/Thy at the same time point by Tukey’s HSD test. **+**, *p* < 0.05 for the comparison between Chitin-treated and control cells by Dunnett’s test.
(TIF)

S4 Fig Expression of miR-155, miR-146a and miR-21 in Chitin- and Chitin+Car/Thy–treated H292 and A549 cells. Expression of miR-155 (A), miR-146a (B) and miR-21 (C) in H292 cells and expression of miR-155 (D), miR-146a (E) and miR-21 (F) in A549 cells in control cells and LPS-treated, poly (I:C)-treated, Car/Thy-treated, chitin-treated and chitin plus Car/Thy-treated cells were determined by qRT-PCR. *, *p* < 0.05 for the comparison between Chitin and Chitin plus Car/Thy at the same time point by Tukey’s HSD test. **+**, *p* < 0.05 for the comparison between Chitin-treated and control cells by Dunnett’s test.
(TIF)

S5 Fig SHIP1 and SOCS1 relative expression in Chitin- and Chitin plus Car/Thy- treated H292 and A549 cells. Expression levels of SHIP1 (A) and SOCS1 (B) in H292 cells and expression of SHIP1 (C) and SOCS1 (D) in A549 cells in control cells and LPS-treated, poly (I:C)-treated, Car/Thy-treated, chitin-treated and chitin plus Car/Thy-treated cells were determined by qRT-PCR. *, *p* < 0.05 for the comparison between Chitin and Chitin plus Car/Thy at the same time point by Tukey’s HSD test. **+**, *p* < 0.05 for the comparison between Chitin-treated and control cells by Dunnett’s test.
(TIF)

## References

[1] Koch BE, Stougaard J, Spaink HP. Keeping track of the growing number of biological functions of chitin and its interaction partners in biomedical research. Glycobiology. 2015;25(5):469–82. 10.1093/glycob/cwv005 25595947 PMC4373397

[2] Van Dyken SJ, Garcia D, Porter P, Huang X, Quinlan PJ, Blanc PD, et al Fungal chitin from asthma-associated home environments induces eosinophilic lung infiltration. J Immunol. 2011;187(5):2261–7. 10.4049/jimmunol.1100972 21824866 PMC3159725

[3] Cartier A, Lehrer SB, Horth-Susin L, Swanson M, Neis B, Howse D, et al Prevalence of crab asthma in crab plant workers in Newfoundland and Labrador. Int J Circumpolar Health. 2004;63 Suppl 2:333–6. . 15736679 10.3402/ijch.v63i0.17930

[4] Jeebhay MF, Robins TG, Miller ME, Bateman E, Smuts M, Baatjies R, et al Occupational allergy and asthma among salt water fish processing workers. American journal of industrial medicine. 2008;51(12):899–910. Epub 2008/08/30. 10.1002/ajim.20635 ; PubMed Central PMCID: PMCPmc2834300. 18726880 PMC2834300

[5] Da Silva CA, Hartl D, Liu W, Lee CG, Elias JA. TLR-2 and IL-17A in chitin-induced macrophage activation and acute inflammation. J Immunol. 2008;181(6):4279–86. 18768886 10.4049/jimmunol.181.6.4279 PMC2577310

[6] Da Silva CA, Chalouni C, Williams A, Hartl D, Lee CG, Elias JA. Chitin is a size-dependent regulator of macrophage TNF and IL-10 production. J Immunol. 2009;182(6):3573–82. Epub 2009/03/07. 10.4049/jimmunol.0802113 . 19265136

[7] Koller B, Muller-Wiefel AS, Rupec R, Korting HC, Ruzicka T. Chitin modulates innate immune responses of keratinocytes. PLoS One. 2011;6(2):e16594 10.1371/journal.pone.0016594 21383982 PMC3044707

[8] Reese TA, Liang HE, Tager AM, Luster AD, Van Rooijen N, Voehringer D, et al Chitin induces accumulation in tissue of innate immune cells associated with allergy. Nature. 2007;447(7140):92–6. 10.1038/nature05746 17450126 PMC2527589

[9] Davies DE. Epithelial barrier function and immunity in asthma. Annals of the American Thoracic Society. 2014;11 Suppl 5:S244–51. 10.1513/AnnalsATS.201407-304AW . 25525727

[10] Erle DJ, Sheppard D. The cell biology of asthma. J Cell Biol. 2014;205(5):621–31. 10.1083/jcb.201401050 24914235 PMC4050726

[11] Van Dyken SJ, Mohapatra A, Nussbaum JC, Molofsky AB, Thornton EE, Ziegler SF, et al Chitin activates parallel immune modules that direct distinct inflammatory responses via innate lymphoid type 2 and gammadelta T cells. Immunity. 2014;40(3):414–24. Epub 2014/03/19. 10.1016/j.immuni.2014.02.003 ; PubMed Central PMCID: PMCPmc4019510. 24631157 PMC4019510

[12] Roy RM, Wuthrich M, Klein BS. Chitin elicits CCL2 from airway epithelial cells and induces CCR2-dependent innate allergic inflammation in the lung. J Immunol. 2012;189(5):2545–52. 10.4049/jimmunol.1200689 22851704 PMC3424300

[13] Lee C, Kolesnik TB, Caminschi I, Chakravorty A, Carter W, Alexander WS, et al Suppressor of cytokine signalling 1 (SOCS1) is a physiological regulator of the asthma response. Clinical and experimental allergy: journal of the British Society for Allergy and Clinical Immunology. 2009;39(6):897–907. Epub 2009/03/25. 10.1111/j.1365-2222.2009.03217.x ; PubMed Central PMCID: PMCPmc3449009. 19309352 PMC3449009

[14] Oh SY, Zheng T, Bailey ML, Barber DL, Schroeder JT, Kim YK, et al Src homology 2 domain-containing inositol 5-phosphatase 1 deficiency leads to a spontaneous allergic inflammation in the murine lung. J Allergy Clin Immunol. 2007;119(1):123–31. 10.1016/j.jaci.2006.08.029 17208593 PMC4757810

[15] Quinn SR, O'Neill LA. A trio of microRNAs that control Toll-like receptor signalling. Int Immunol. 2011;23(7):421–5. Epub 2011/06/10. 10.1093/intimm/dxr034 . 21652514

[16] Liu J, Drescher KM, Chen XM. MicroRNAs and Epithelial Immunity. Int Rev Immunol. 2009;28(3–4):139–54. 10.1080/08830180902943058 19811319 PMC2764336

[17] Perry MM, Adcock IM, Chung KF. Role of microRNAs in allergic asthma: present and future. Curr Opin Allergy Clin Immunol. 2015;15(2):156–62. 10.1097/ACI.0000000000000147 . 25961389

[18] Billeter AT, Hellmann J, Roberts H, Druen D, Gardner SA, Sarojini H, et al MicroRNA-155 potentiates the inflammatory response in hypothermia by suppressing IL-10 production. FASEB J. 2014;28(12):5322–36. 10.1096/fj.14-258335 25231976 PMC4232280

[19] de Cassia da Silveira e Sa R, Andrade LN, de Sousa DP. A review on anti-inflammatory activity of monoterpenes. Molecules. 2013;18(1):1227–54. Epub 2013/01/22. 10.3390/molecules18011227 . 23334570 PMC6269770

[20] El-Sayed EM, Abd-Allah AR, Mansour AM, El-Arabey AA. Thymol and carvacrol prevent cisplatin-induced nephrotoxicity by abrogation of oxidative stress, inflammation, and apoptosis in rats. J Biochem Mol Toxicol. 2015;29(4):165–72. 10.1002/jbt.21681 . 25487789

[21] Khosravi AR, Shokri H, Minooeianhaghighi M. Inhibition of aflatoxin production and growth of Aspergillus parasiticus by Cuminum cyminum, Ziziphora clinopodioides, and Nigella sativa essential oils. Foodborne Pathog Dis. 2011;8(12):1275–80. 10.1089/fpd.2011.0929 . 21861703

[22] Boskabady MH, Jalali S. Effect of carvacrol on tracheal responsiveness, inflammatory mediators, total and differential WBC count in blood of sensitized guinea pigs. Exp Biol Med (Maywood). 2013;238(2):200–8. 10.1177/1535370212474604 . 23576802

[23] Mora-Montes HM, Netea MG, Ferwerda G, Lenardon MD, Brown GD, Mistry AR, et al Recognition and blocking of innate immunity cells by Candida albicans chitin. Infect Immun. 2011;79(5):1961–70. 10.1128/IAI.01282-10 21357722 PMC3088140

[24] Alvarez FJ. The effect of chitin size, shape, source and purification method on immune recognition. Molecules. 2014;19(4):4433–51. 10.3390/molecules19044433 . 24727416 PMC6271096

[25] Gholijani N, Gharagozloo M, Farjadian S, Amirghofran Z. Modulatory effects of thymol and carvacrol on inflammatory transcription factors in lipopolysaccharide-treated macrophages. J Immunotoxicol. 2015:1–8. 10.3109/1547691X.2015.1029145 . 25812626

[26] Llana-Ruiz-Cabello M, Gutiérrez-Praena D, Pichardo S, Moreno FJ, Bermúdez JM, Aucejo S, et al Cytotoxicity and morphological effects induced by carvacrol and thymol on the human cell line Caco-2. Food Chem Toxicol. 2014;64:281–90. 10.1016/j.fct.2013.12.005 24326232

[27] Livak KJ, Schmittgen TD. Analysis of relative gene expression data using real-time quantitative PCR and the 2(-Delta Delta C(T)) Method. Methods. 2001;25(4):402–8. 10.1006/meth.2001.1262 . 11846609

[28] Schneider CA, Rasband WS, Eliceiri KW. NIH Image to ImageJ: 25 years of image analysis. Nat Methods. 2012;9(7):671–5. . 22930834 10.1038/nmeth.2089 PMC5554542

[29] Bouhenna M, Salah R, Bakour R, Drouiche N, Abdi N, Grib H, et al Effects of chitin and its derivatives on human cancer cells lines. Environmental Science and Pollution Research. 2015;22(20):15579–86. 10.1007/s11356-015-4712-3 26013739

[30] Matsui M, Ono L, Akcelrud L. Chitin/polyurethane networks and blends: evaluation of biological application. Polymer Testing. 2012;31(1):191–6.

[31] Shokri H, Asadi F, Khosravi AR. Isolation of beta-glucan from the cell wall of Saccharomyces cerevisiae. Natural product research. 2008;22(5):414–21. 10.1080/14786410701591622 . 18404561

[32] Goldman DL, Vicencio AG. The chitin connection. mBio. 2012;3(2). 10.1128/mBio.00056-12 22448043 PMC3315704

[33] Roy RM, Klein BS. Fungal glycan interactions with epithelial cells in allergic airway disease. Curr Opin Microbiol. 2013;16(4):404–8. 10.1016/j.mib.2013.03.004 23602359 PMC3893682

[34] Kim LK, Morita R, Kobayashi Y, Eisenbarth SC, Lee CG, Elias J, et al AMCase is a crucial regulator of type 2 immune responses to inhaled house dust mites. Proc Natl Acad Sci U S A. 2015;112(22):E2891–9. Epub 2015/06/04. 10.1073/pnas.1507393112 ; PubMed Central PMCID: PMCPmc4460510. 26038565 PMC4460510

[35] Wiesner DL, Specht CA, Lee CK, Smith KD, Mukaremera L, Lee ST, et al Chitin recognition via chitotriosidase promotes pathologic type-2 helper T cell responses to cryptococcal infection. PLoS Pathog. 2015;11(3):e1004701 10.1371/journal.ppat.1004701 25764512 PMC4357429

[36] Yasuda K, Muto T, Kawagoe T, Matsumoto M, Sasaki Y, Matsushita K, et al Contribution of IL-33-activated type II innate lymphoid cells to pulmonary eosinophilia in intestinal nematode-infected mice. Proc Natl Acad Sci U S A. 2012;109(9):3451–6. 10.1073/pnas.1201042109 22331917 PMC3295287

[37] Mohapatra A, Van Dyken SJ, Schneider C, Nussbaum JC, Liang HE, Locksley RM. Group 2 innate lymphoid cells utilize the IRF4-IL-9 module to coordinate epithelial cell maintenance of lung homeostasis. Mucosal Immunol. 2016;9(1):275–86. 10.1038/mi.2015.59 26129648 PMC4698110

[38] Phipps S, Lam CE, Foster PS, Matthaei KI. The contribution of toll-like receptors to the pathogenesis of asthma. Immunol Cell Biol. 2007;85(6):463–70. 10.1038/sj.icb.7100104 . 17680012

[39] Wang JW, Li K, Hellermann G, Lockey RF, Mohapatra S, Mohapatra S. Regulating the Regulators: microRNA and Asthma. World Allergy Organ J. 2011;4(6):94–103. 10.1097/WOX.0b013e31821d1186 23282474 PMC3651079

[40] Sessa R, Hata A. Role of microRNAs in lung development and pulmonary diseases. Pulm Circ. 2013;3(2):315–28. 10.4103/2045-8932.114758 24015331 PMC3757825

[41] Wu XB, Wang MY, Zhu HY, Tang SQ, You YD, Xie YQ. Overexpression of microRNA-21 and microRNA-126 in the patients of bronchial asthma. Int J Clin Exp Med. 2014;7(5):1307–12. 24995087 PMC4073748

[42] Lu TX, Munitz A, Rothenberg ME. MicroRNA-21 is up-regulated in allergic airway inflammation and regulates IL-12p35 expression. J Immunol. 2009;182(8):4994–5002. Epub 2009/04/04. 10.4049/jimmunol.0803560 ; PubMed Central PMCID: PMCPmc4280862. 19342679 PMC4280862

[43] Lampe WR, Park J, Fang S, Crews AL, Adler KB. Calpain and MARCKS protein regulation of airway mucin secretion. Pulm Pharmacol Ther. 2012;25(6):427–31. 10.1016/j.pupt.2012.06.003 22710197 PMC3486950

[44] Park J, Fang S, Crews AL, Lin KW, Adler KB. MARCKS regulation of mucin secretion by airway epithelium in vitro: interaction with chaperones. Am J Respir Cell Mol Biol. 2008;39(1):68–76. 10.1165/rcmb.2007-0139OC 18314541 PMC2438449

[45] Rebane A, Akdis CA. MicroRNAs in allergy and asthma. Current allergy and asthma reports. 2014;14(4):424 10.1007/s11882-014-0424-x . 24504527

[46] Vigorito E, Kohlhaas S, Lu D, Leyland R. miR-155: an ancient regulator of the immune system. Immunol Rev. 2013;253(1):146–57. 10.1111/imr.12057 . 23550644

[47] Lind EF, Ohashi PS. Mir-155, a central modulator of T-cell responses. Eur J Immunol. 2014;44(1):11–5. . 24571026 10.1002/eji.201343962

[48] Huang X, Shen Y, Liu M, Bi C, Jiang C, Iqbal J, et al Quantitative proteomics reveals that miR-155 regulates the PI3K-AKT pathway in diffuse large B-cell lymphoma. Am J Pathol. 2012;181(1):26–33. 10.1016/j.ajpath.2012.03.013 22609116 PMC3388146

[49] Cheung ST, So EY, Chang D, Ming-Lum A, Mui AL. Interleukin-10 inhibits lipopolysaccharide induced miR-155 precursor stability and maturation. PLoS One. 2013;8(8):e71336 10.1371/journal.pone.0071336 23951138 PMC3741136

[50] Gholijani N, Gharagozloo M, Kalantar F, Ramezani A, Amirghofran Z. Modulation of Cytokine Production and Transcription Factors Activities in Human Jurkat T Cells by Thymol and Carvacrol. Adv Pharm Bull. 2015;5(Suppl 1):653–60. 10.15171/apb.2015.089 26793612 PMC4708037

[51] Liang D, Li F, Fu Y, Cao Y, Song X, Wang T, et al Thymol inhibits LPS-stimulated inflammatory response via down-regulation of NF-kappaB and MAPK signaling pathways in mouse mammary epithelial cells. Inflammation. 2014;37(1):214–22. 10.1007/s10753-013-9732-x . 24057926

[52] Juergens U, Dethlefsen U, Steinkamp G, Gillissen A, Repges R, Vetter H. Anti-inflammatory activity of 1.8-cineol (eucalyptol) in bronchial asthma: a double-blind placebo-controlled trial. Respir Med. 2003;97(3):250–6. 12645832 10.1053/rmed.2003.1432

[53] Morice A, Marshall A, Higgins K, Grattan T. Effect of inhaled menthol on citric acid induced cough in normal subjects. Thorax. 1994;49(10):1024–6. 7974298 10.1136/thx.49.10.1024 PMC475243

[54] Tamaoki J, Chiyotani A, Sakai A, Takemura H, Konno K. Effect of menthol vapour on airway hyperresponsiveness in patients with mild asthma. Respir Med. 1995;89(7):503–4. 7480981 10.1016/0954-6111(95)90127-2

[55] Pirigharnaei M, Zare S, Heidary R, Khara J, EmamaliSabzi R, Kheiry F. The essential oils compositions of Iranian Oregano (Origanum vulgareL.) populations in field and provenance from Piranshahr district, West Azarbaijan province, Iran. Avicenna Journal of Phytomedicine. 2011;1(2):106–14.

[56] Karpouhtsis I, Pardali E, Feggou E, Kokkini S, Scouras ZG, Mavragani-Tsipidou P. Insecticidal and genotoxic activities of oregano essential oils. J Agric Food Chem. 1998;46(3):1111–5.

[57] Tisserand R, Young R. Essential oil safety: a guide for health care professionals: Elsevier Health Sciences; 2013.

