# Supplementary figures and images for: Chitin-Induced Airway Epithelial Cell Innate Immune Responses Are Inhibited by Carvacrol/Thymol
Source: PLoS One. 2016 Jul 27;11(7):e0159459. doi: 10.1371/journal.pone.0159459 (PMC4962986; doi:10.1371/journal.pone.0159459)

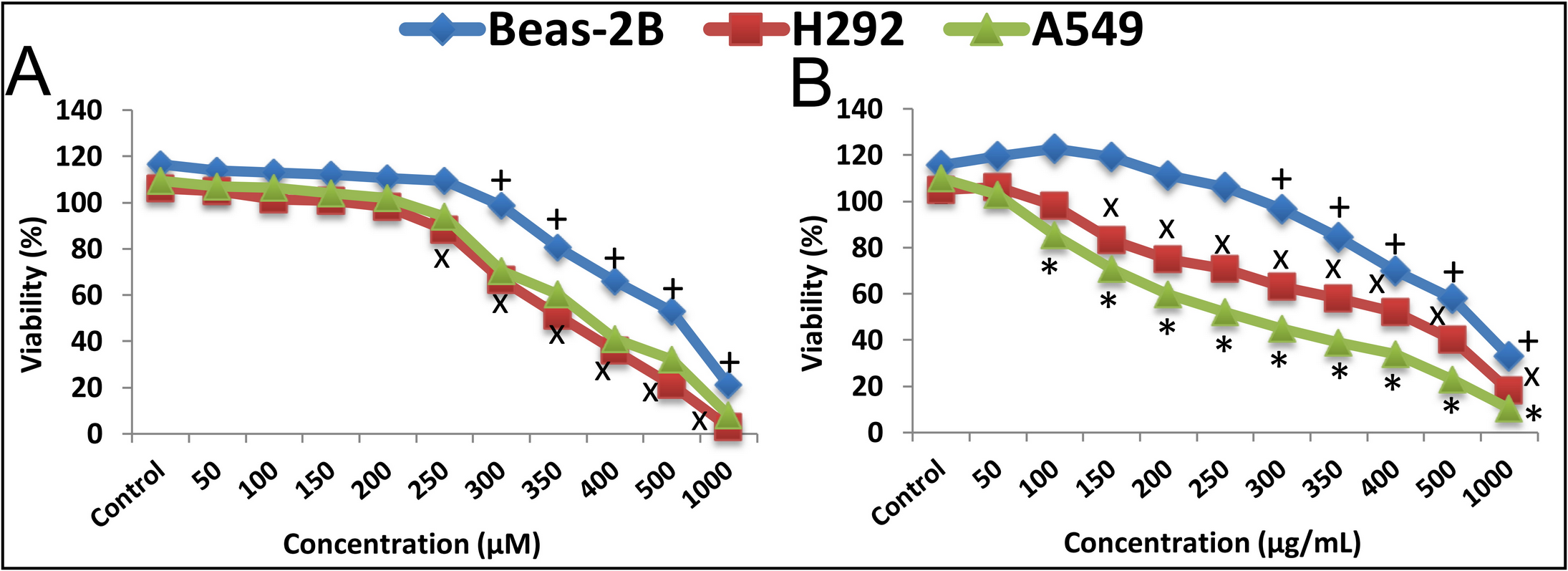

Supplement: S1 Fig — Viability of cells treated with Car/Thy (A) and chitin (B) at 50–1000 μM and 50–1000 μg/mL, respectively were determined by MTT assay after 24 h incubation. +, X and *, p < 0.05 are for the comparison between control cells and BEAS-2B, H292 and A549 cells, respectively by Dunnett’s test. Nearly 100% of cells treated with Triton-X100 (as positive control) were dead (not viable). (TIF) [file pone.0159459.s001.tif]

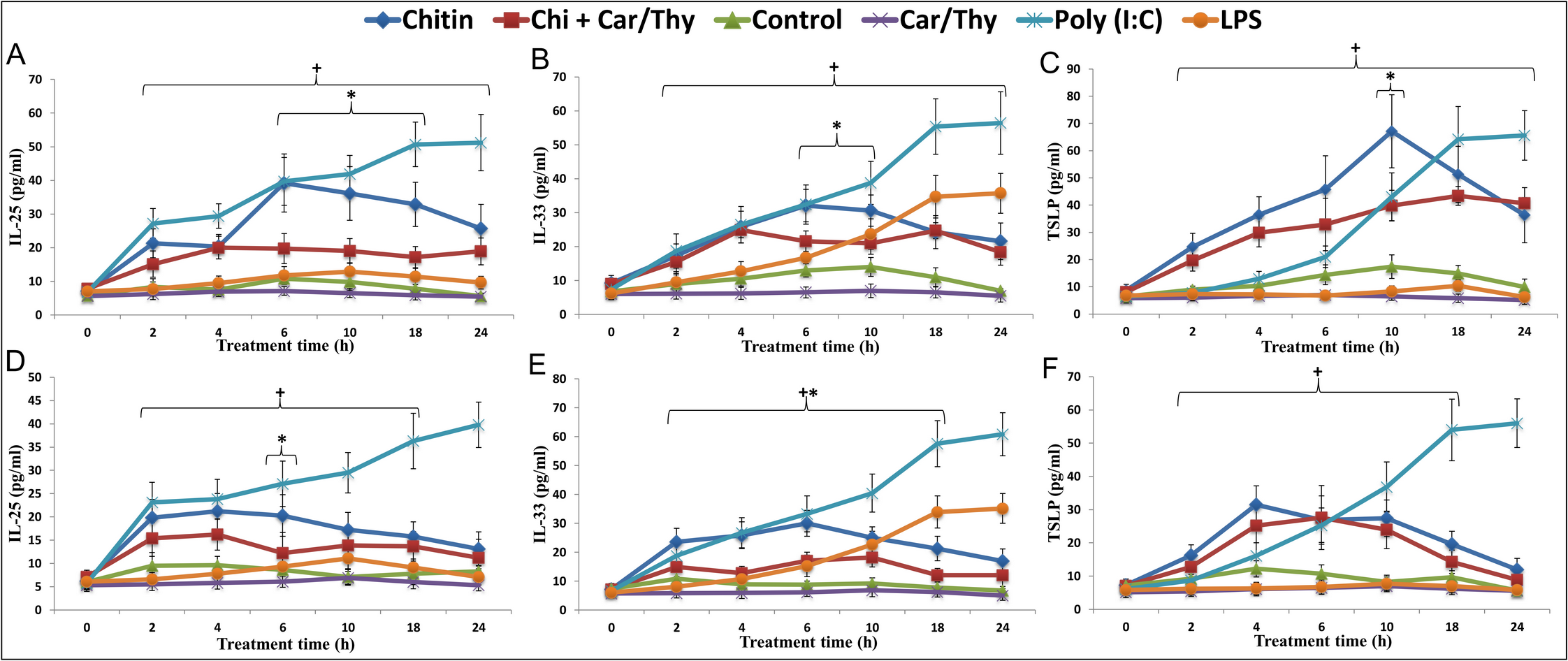

Supplement: S2 Fig — Levels of IL-25 (A), IL-33 (B), and TSLP (C) in H292 cells and levels of IL-25 (D), IL-33 (E), and TSLP (F) in A549 cells in supernatant from control, LPS-treated, poly (I:C)-treated, Car/Thy-treated, chitin-treated and chitin plus Car/Thy-treated cells were determined by ELISA. *, p < 0.05 for the comparison between Chitin and Chitin plus Car/Thy at the same time point by Tukey’s HSD test. +, p < 0.05 for the comparison between Chitin-treated and control cells by Dunnett’s test. (TIF) [file pone.0159459.s002.tif]

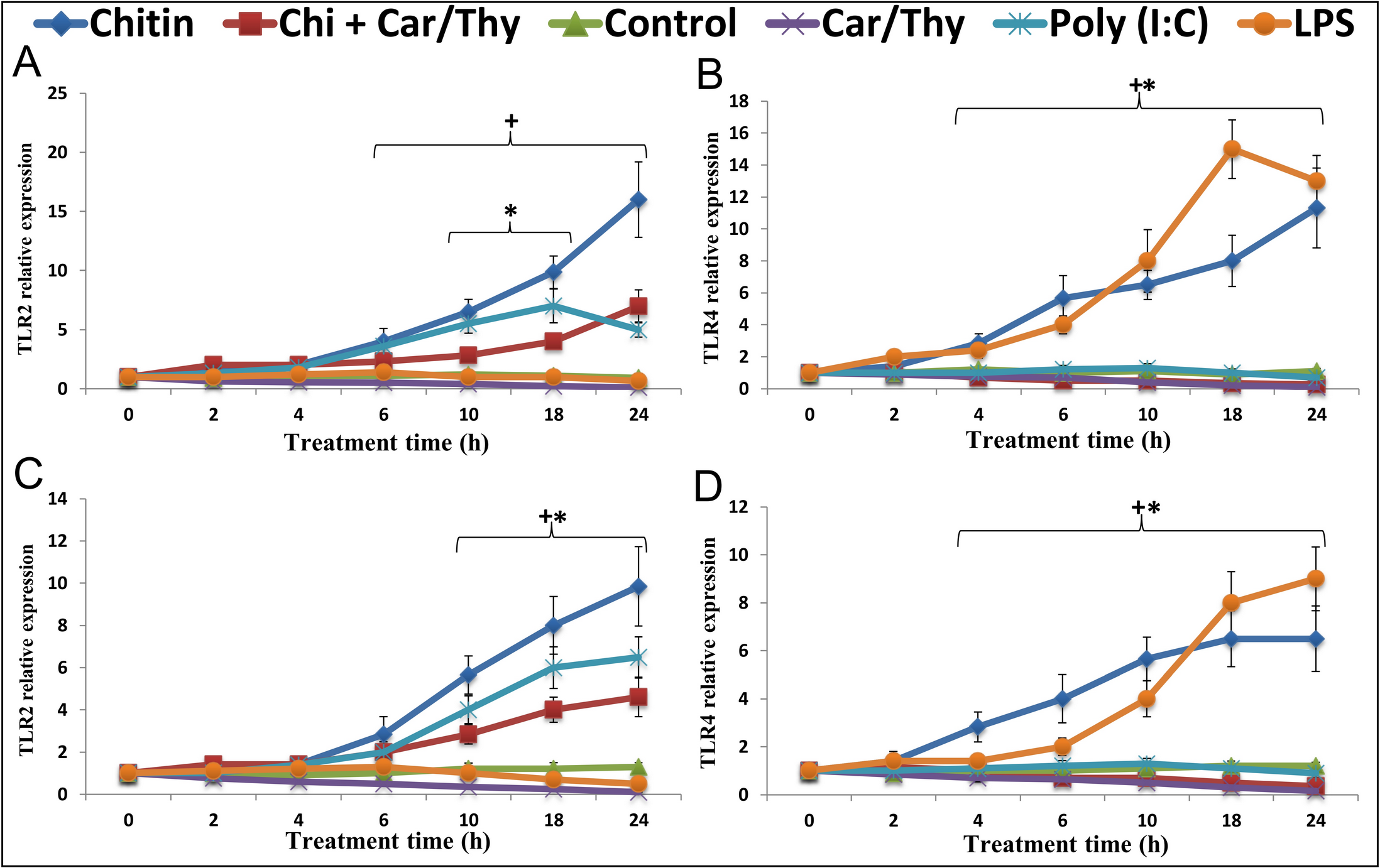

Supplement: S3 Fig — mRNA Levels of TLR2 (A) and TLR4 (B) in H292 cells and TLR2 (C) and TLR4 (D) in A549 cells in control cells and LPS-treated, poly (I:C)-treated, Car/Thy-treated, chitin-treated and chitin plus Car/Thy-treated cells were determined by qRT-PCR. *, p < 0.05 for the comparison between Chitin and Chitin plus Car/Thy at the same time point by Tukey’s HSD test. +, p < 0.05 for the comparison between Chitin-treated and control cells by Dunnett’s test. (TIF) [file pone.0159459.s003.tif]

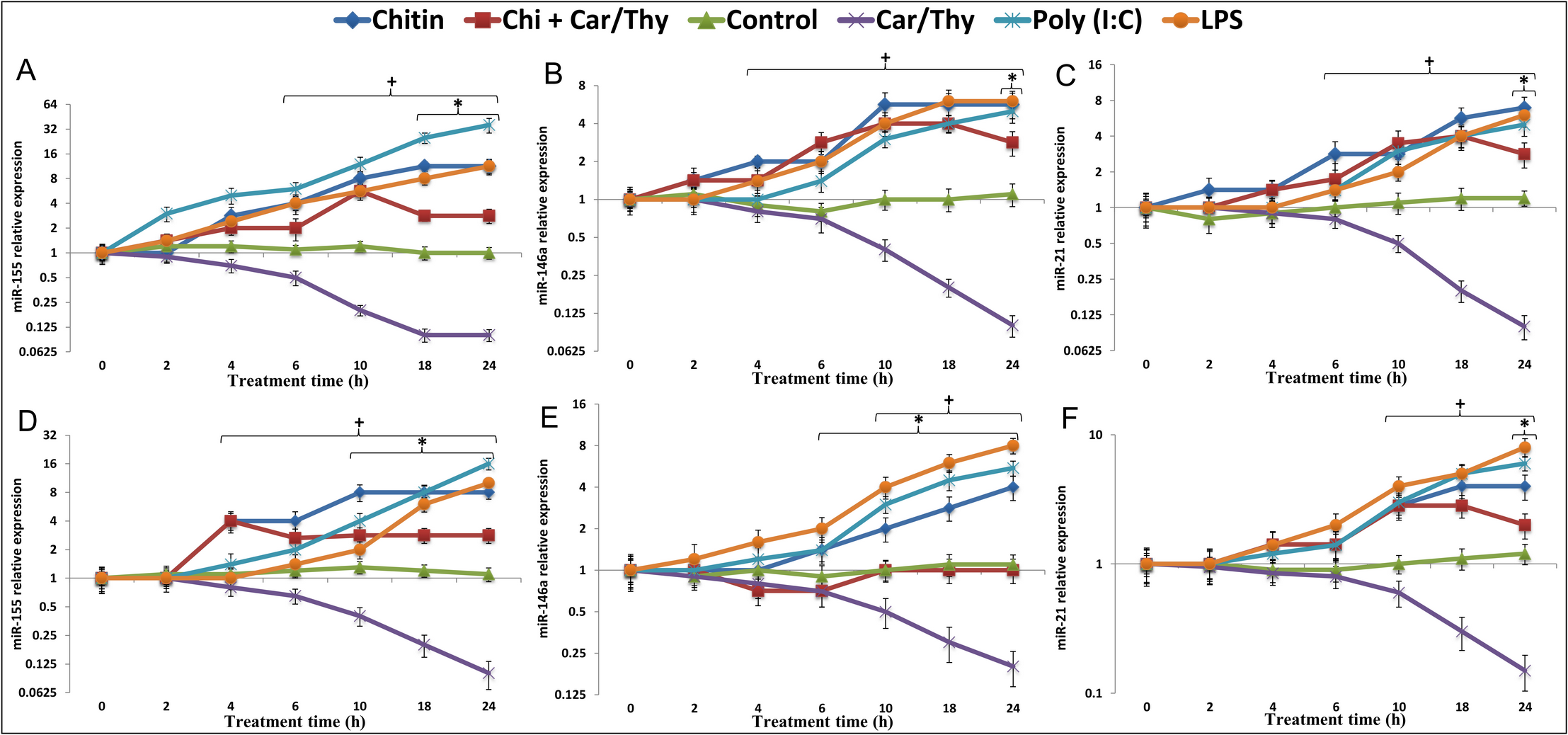

Supplement: S4 Fig — Expression of miR-155 (A), miR-146a (B) and miR-21 (C) in H292 cells and expression of miR-155 (D), miR-146a (E) and miR-21 (F) in A549 cells in control cells and LPS-treated, poly (I:C)-treated, Car/Thy-treated, chitin-treated and chitin plus Car/Thy-treated cells were determined by qRT-PCR. *, p < 0.05 for the comparison between Chitin and Chitin plus Car/Thy at the same time point by Tukey’s HSD test. +, p < 0.05 for the comparison between Chitin-treated and control cells by Dunnett’s test. (TIF) [file pone.0159459.s004.tif]

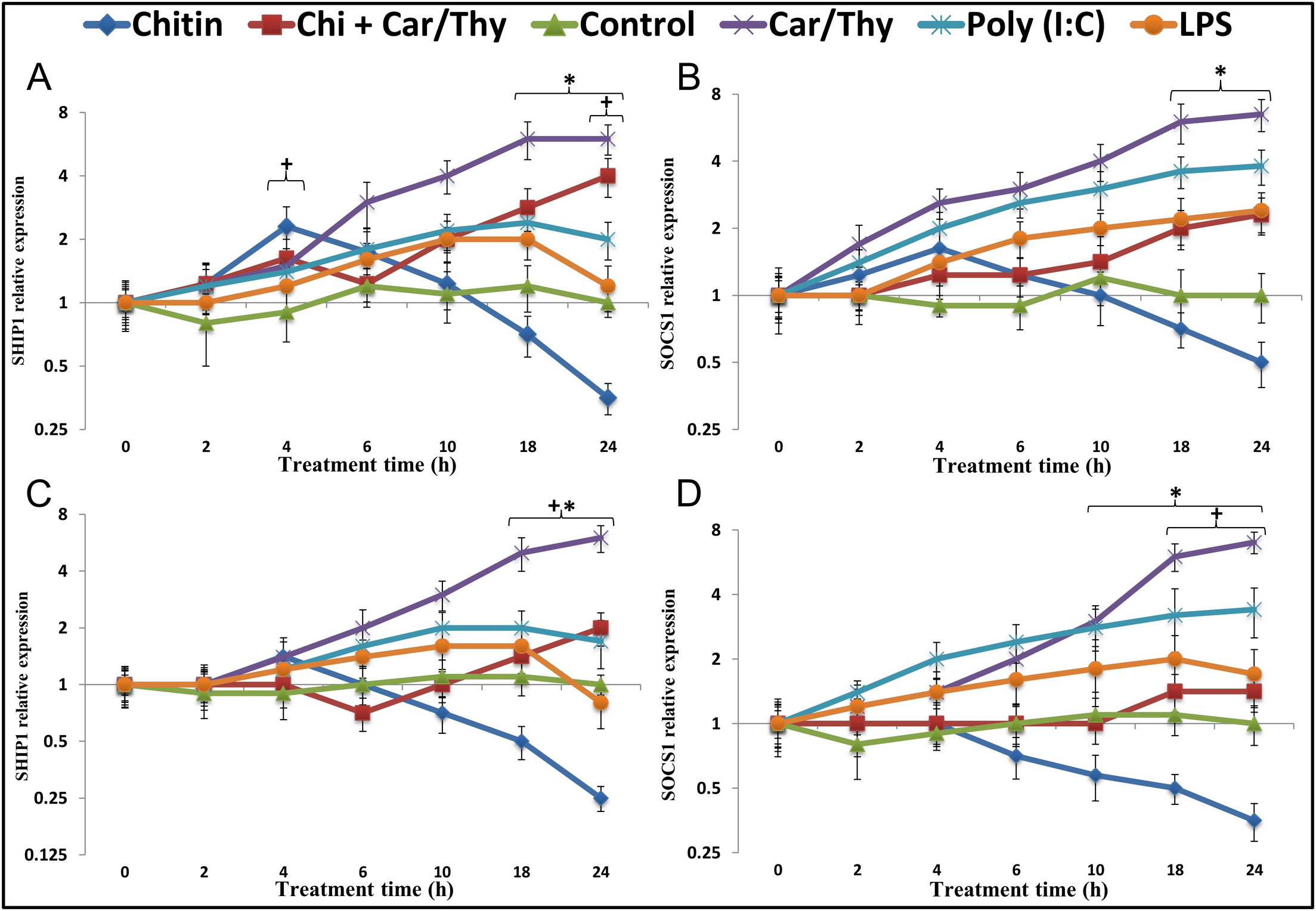

Supplement: S5 Fig — Expression levels of SHIP1 (A) and SOCS1 (B) in H292 cells and expression of SHIP1 (C) and SOCS1 (D) in A549 cells in control cells and LPS-treated, poly (I:C)-treated, Car/Thy-treated, chitin-treated and chitin plus Car/Thy-treated cells were determined by qRT-PCR. *, p < 0.05 for the comparison between Chitin and Chitin plus Car/Thy at the same time point by Tukey’s HSD test. +, p < 0.05 for the comparison between Chitin-treated and control cells by Dunnett’s test. (TIF) [file pone.0159459.s005.tif]
